# Supplementary material for: Asymmetric CRISPR enabling cascade signal amplification for nucleic acid detection by competitive crRNA
Source: Nat Commun. 2023 Nov 18;14:7504. doi: 10.1038/s41467-023-43389-7 (PMC10657364; doi:10.1038/s41467-023-43389-7)
Supplement: Supplementary file 1 — Supplementary Information [file 41467_2023_43389_MOESM1_ESM.pdf]

Supplementary Information for

Asymmetric CRISPR Enabling Cascade Signal Amplification for Nucleic Acid Detection by Competitive crRNA

Jeong Moon[1], *Changchun Liu*[1] *

[1] *Department of Biomedical Engineering, University of Connecticut Health Center, Farmington, Connecticut 06032, United States*

* **Corresponding author**
Dr. Changchun Liu
Department of Biomedical Engineering
University of Connecticut Health Center
263 Farmington Avenue
Farmington, CT 06030
Phone: (860)-679-2565
E-mail: chaliu@uchc.edu

Supplementary Table 1. Summary of the ratio of positive tests in asymmetric CRISPR assay to detect the target miRNA. The probit analysis demonstrates that the 95% detection limit of the asymmetric CRISPR assay is 856 aM.

Target miRNA concentration	No. of positive tests / No. of total tests
1 fM	15/15
500 aM	10/15
200 aM	7/15
100 aM	0/15
50 aM	0/15

Supplementary Table 2. Comparison of asymmetric CRISPR assay with other miRNA detection methods using CRISPR/Cas12a system

**Method**	**Mechanism**	**One-pot reaction**	**# of major components**	**LOD**	**Ref**
PCDetection	polyA-tailing + cDNA synthesis + Recombinase polymerase amplification (RPA) + Cas12a	No, 3 steps	4 enzymes, 3 probes	miR-299: 50 fM	[1]
CRISPR-HCR	Hybridization chain reaction (HCR) + Cas12a	No, 2 steps	1 enzyme, 3 probes	miR-21: 1 fM	[2]
EXTRA-CRISPR	Rolling-circle amplification (RCA) + Cas12a	Yes	3 enzymes, 3 probes	miR-21: 1.64 fM; miR-196a: 1.35 fM	[3]
CRISPR-CHA	Catalytic hairpin assembly (CHA) + Cas12a	No, 2 steps	1 enzyme, 2 probes	miR-141: 0.14 fM; miR-155: 0.15 fM	[4]
ccTdT-Cas12a	Click chemistry ligation + Magnetic separation + polyT-tailing + Cas12a	No, 4 steps	2 enzymes, 2 probes	miR-21: 88 fM	[5]
CAL-LAMP	Ligation + Loop-mediated Isothermal Amplification (LAMP) + Cas12a	No, 3 steps	3 enzymes, 6 probes	Let-7a: 0.1 fM	[6]
Cas12a-enhanced RCA	Ligation + Rolling-circle amplification (RCA) + Cas12a	No, 3 steps	3 enzymes, 3 probes	miR-21: 34.7 fM	[7]
**Asymmetric CRISPR**	**Cascade signal amplification based on the competitive reaction between Full-sized crRNA and split crRNA**	**Yes**	**1 enzyme, Split crRNA, 1 activator**	**miR19a: 856 aM**	**This study**

Supplementary Table 3. The estimated cost of the asymmetric CRISPR assay

	**Volume per reaction**	**Price per kit**	**Volume per kit**	**Cost per reaction**
LbCas12	2 μL	$ 263	20 μL	$ 0.263
crRNA	0.8 μL	$ 145	100 μL	$ 0.012
FQ reporter	2 μL	$ 588	610 μL	$ 0.193
split handle	0.2 μL	$ 188	150 μL	$ 0.0025
split spacer	0.2 μL	$ 188	150 μL	$ 0.0025
DNA probe	0.4 μL	$ 14	100 μL	$ 0.00056

Total: $ 0.47

Supplementary Table 4. Oligonucleotide sequences

Name	Sequence (5'→3')	bp
RD crRNA 8	/AltR1/rUrA rArUrU rUrCrU rArCrU rArArG rUrGrU rArGrA rUrUrU rCrUrU rCrUrA rUrCrA rGrUrU rUrUrG rCrArU rA/AltR2/	42
RD crRNA 9	/AltR1/rUrA rArUrU rUrCrU rArCrU rArArG rUrGrU rArGrA rUrArU rUrCrU rUrCrU rArUrC rArGrU rUrUrU rGrCrA rU/AltR2/	42
RD crRNA 10	/AltR1/rUrA rArUrU rUrCrU rArCrU rArArG rUrGrU rArGrA rUrCrA rUrUrC rUrUrC rUrArU rCrArG rUrUrU rUrGrC rA/AltR2/	42
RD crRNA 11	/AltR1/rUrA rArUrU rUrCrU rArCrU rArArG rUrGrU rArGrA rUrArC rArUrU rCrUrU rCrUrA rUrCrA rGrUrU rUrUrG rC/AltR2/	42
RD crRNA 12	/AltR1/rUrA rArUrU rUrCrU rArCrU rArArG rUrGrU rArGrA rUrCrA rCrArU rUrCrU rUrCrU rArUrC rArGrU rUrUrU rG/AltR2/	42
RD crRNA 13	/AltR1/rUrA rArUrU rUrCrU rArCrU rArArG rUrGrU rArGrA rUrArC rArCrA rUrUrC rUrUrC rUrArU rCrArG rUrUrU rU/AltR2/	42
DD crRNA 8	/AltR1/rUrA rArUrU rUrCrU rArCrU rArArG rUrGrU rArGrA rUrUrU rUrGrC rArCrA rUrGrA rGrUrC rGrUrA rUrUrA rU/AltR2/	42
DD crRNA 9	/AltR1/rUrA rArUrU rUrCrU rArCrU rArArG rUrGrU rArGrA rUrArU rUrUrG rCrArC rArUrG rArGrU rCrGrU rArUrU rA/AltR2/	42
DD crRNA 10	/AltR1/rUrA rArUrU rUrCrU rArCrU rArArG rUrGrU rArGrA rUrGrA rUrUrU rGrCrA rCrArU rGrArG rUrCrG rUrArU rU/AltR2/	42
DD crRNA 11	/AltR1/rUrA rArUrU rUrCrU rArCrU rArArG rUrGrU rArGrA rUrArG rArUrU rUrGrC rArCrA rUrGrA rGrUrC rGrUrA rU/AltR2/	42
DD crRNA 12	/AltR1/rUrA rArUrU rUrCrU rArCrU rArArG rUrGrU rArGrA rUrUrA rGrArU rUrUrG rCrArC rArUrG rArGrU rCrGrU rA/AltR2/	42
DD crRNA 13	AltR1/rUrA rArUrU rUrCrU rArCrU rArArG rUrGrU rArGrA rUrArU rArGrA rUrUrU rGrCrA rCrArU rGrArG rUrCrG rU/AltR2/	42
miR-19a	UGU GCA AAU CUA UGC AAA ACU GA	23
miR-19a DNA	TGT GCA AAT CTA TGC AAA ACT GA	23

Let7a crRNA	/AltR1/rUrA rArUrU rUrCrU rArCrU rArArG rUrGrU rArGrA rUrCrA rCrArU rUrCrU rUrCrU rArArA rCrUrA rUrArC rA/AltR2/	42
Let-7a	UGA GGU AGU AGG UUG UAU AGU U	22
Let-7b	rUrGrA rGrGrU rArGrU rArGrG rUrUrG rUrGrU rGrGrU rU	22
Let-7c	rUrGrA rGrGrU rArGrU rArGrG rUrUrG rUrArU rGrGrU rU	22
miR-21	UAG CUU AUC AGA CUG AUG UUG A	22
miR-155	UUA AUG CUA AUC GUG AUA GGG GUU	24
miR-122	UGG AGU GUG ACA AUG GUG UUU G	22
Universal DNA probe	TAG AAG AAT GTG TAA GTA TAA TAC GAC TCA	30
FAM reporter	/56-FAM/TT ATT /3IABkFQ/	5
RD DNA target (Long ssDNA)	TGT GCA AAT CTA TGC AAA ACT GAT AGA AGA ATG TGT AAG TAT AAT ACG ACT CA	53
crRNA handle	rUrArA rUrUrU rCrUrA rCrUrA rArGrU rGrUrA rGrArU	21
crRNA spacer	rUrGrA rGrUrC rGrUrA rUrUrA rUrArC rUrUrA rCrArC	21
Target-specific full-sized crRNA	/AltR1/rUrA rArUrU rUrCrU rArCrU rArArG rUrGrU rArGrA rUrUrG rArGrU rCrGrU rArUrU rArUrA rCrUrU rArCrA rC/AltR2/	42
Competitor full-sized crRNA	/AltR1/rUrA rArUrU rUrCrU rArCrU rArArG rUrGrU rArGrA rUrCrA rCrArU rUrCrU rUrCrU rArUrC rArGrU rUrUrU rG/AltR2/	42
Competitor split crRNA spacer	rArArC rUrArU rArCrA rArCrC rUrArC rUrArC rCrUrC rA	22
Split crRNA target ssDNA	TGA GGT AGT AGG TTG TAT AGT T	22
Cy5-full sized crRNA	/5Cy5/rUrA rArUrU rUrCrU rArCrU rArArG rUrGrU rArGrA rUrCrA rCrArU rUrCrU rUrCrU rArUrC rArGrU rUrUrU rG	42

FAM-split crRNA handle	/56-FAM/rUrA rArUrU rUrCrU rArCrU rArArG rUrGrU rArGrA rU	21
Cy5-full-T	/5Cy5/TG TGC AAA TCT ATG CAA AAC TGA TAG AAG AAT GTG TAA GTA TAA TAC GAC TCA	53
FAM-split-T	/56-FAM/AA ATG AGG TAG TAG GTT GTA TAG TT	25
Cy3-non-target	/5Cy3/GA TGT ACA AAT ATC CAG TGG AAC TTC ACT TTT G	33

Supplementary Table 5. Bladder cancer patient sample information. Age range is 45-78.

	Gender	Diagnosis
1	M	High-grade T2-2 papillary
2	F	Focal papillary and focal *in situ* urothelial carcinoma, grade 3/3
3	M	High-grade urothelial papillary transitional cell carcinoma, grade 3/3
4	M	Exophytic, papillary transitional cell carcinoma, grade 1 with scattered foci grade 2/3
5	M	High-grade urothelial (transitional cell) papillary carcinoma
6	M	High-grade invasive urothelial carcinoma
7	M	Focus of high-grade urothelial carcinoma
8	M	Urothelial (transitional cell) carcinoma, foci of high grade within background of low grade
9	M	Urothelial (transitional cell) carcinoma, pTa, grade 2/3
10	M	High-grade urothelial carcinoma, pT1

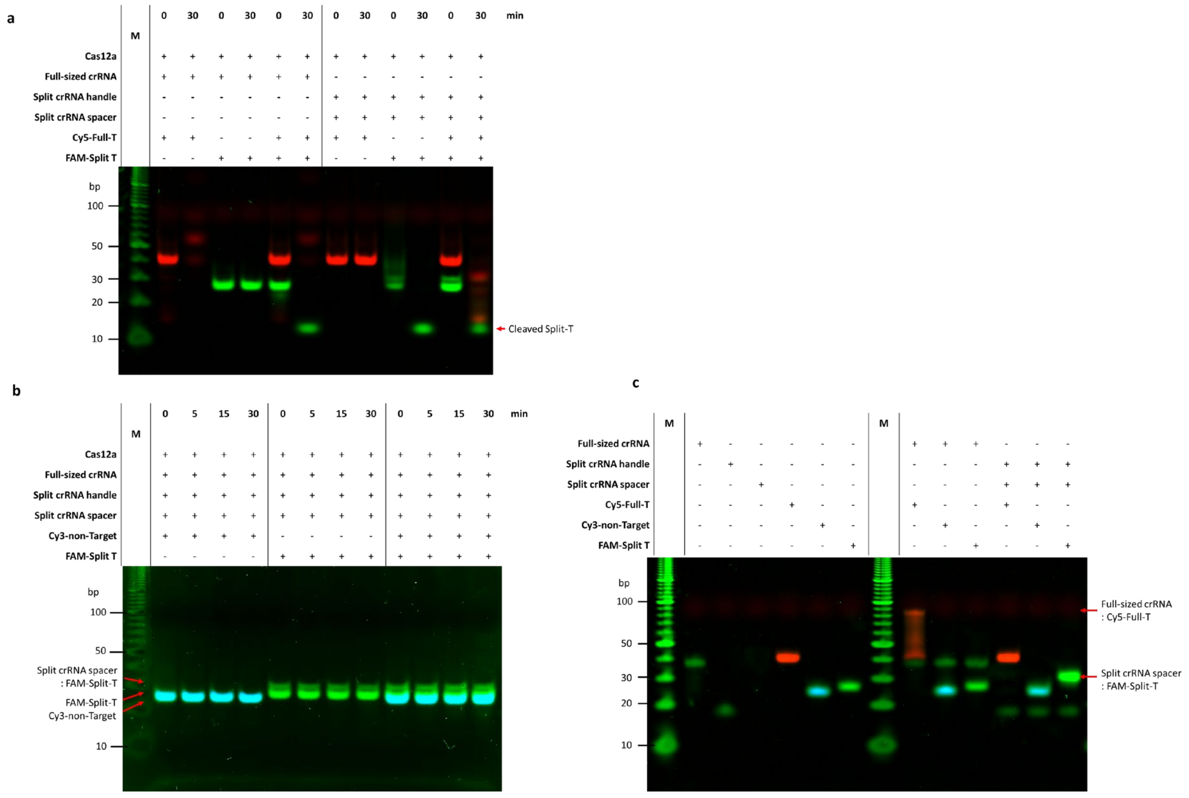

Supplementary Figure 1. Gel images measured after SYBR gold staining**. (a)** Cas12a were assembled with full-sized crRNA and split crRNA, respectively, and they were mixed with various combinations of Cy5-full-T and FAM-split-T. Gel was measured by Cy5- and Alexa488-filter and the gel images were merged. Red and green colors represent Cy5- and FAM/SYBR gold-signal, respectively. **(b)** Cas12a, full-sized crRNA, and split crRNA were mixed with various combinations of Cy3-non-target and FAM-split-T. Gel was measured by Cy3- and Alexa488-filter and the gel images were merged. Blue and green colors represent Cy3- and FAM/SYBR gold-signal, respectively. [LbCas12a] = 100 nM, [Full-sized crRNA] = 40 nM, [Split crRNA handle and spacer] = 10 nM each, [Cy5-full-T] = 100 nM, [Cy3-non-target] = 100 nM, and [FAM-Split-T] = 50 nM. **(c)** Gel was measured by Cy5-, Cy3-, and Alexa488-filter, and the gel images were merged. Red, blue, and green colors represent Cy5-, Cy3-, and FAM/SYBR gold-signal, respectively. [All nucleotides] = 100 nM. M = marker. These experiments were performed twice. Source data are provided as a Source Data file.

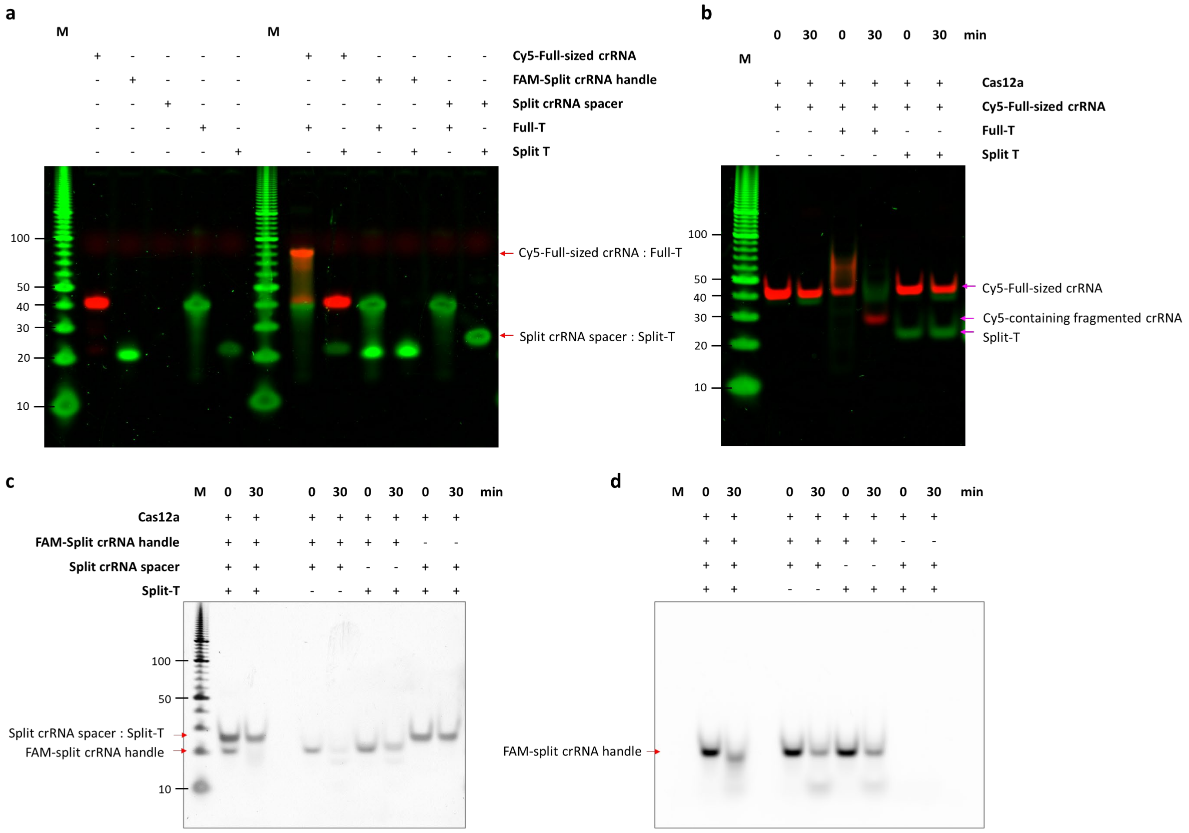

Supplementary Figure 2. (a, b) Gel image measured after SYBR gold staining**.** Gel was measured by Cy5- and Alexa488-filter and the gel images were merged. Red and green colors represent Cy5- and FAM/SYBR gold-signal, respectively. **(c)** Gel image measured after SYBR gold staining. **(d)** Gel image measured before SYBR gold staining. The reduced FAM fluorescence signal after the CRISPR-Cas12a reaction in the absence of split-T indicates pre-crRNA processing. [Cas12a] = 200 nM, [Cy5-full-sized crRNA, FAM-split crRNA handle and split crRNA spacer] = 100 nM, [Full-T] = 100 nM, and [Split-T] = 50 nM. M = marker. These experiments were performed twice. Source data are provided as a Source Data file.

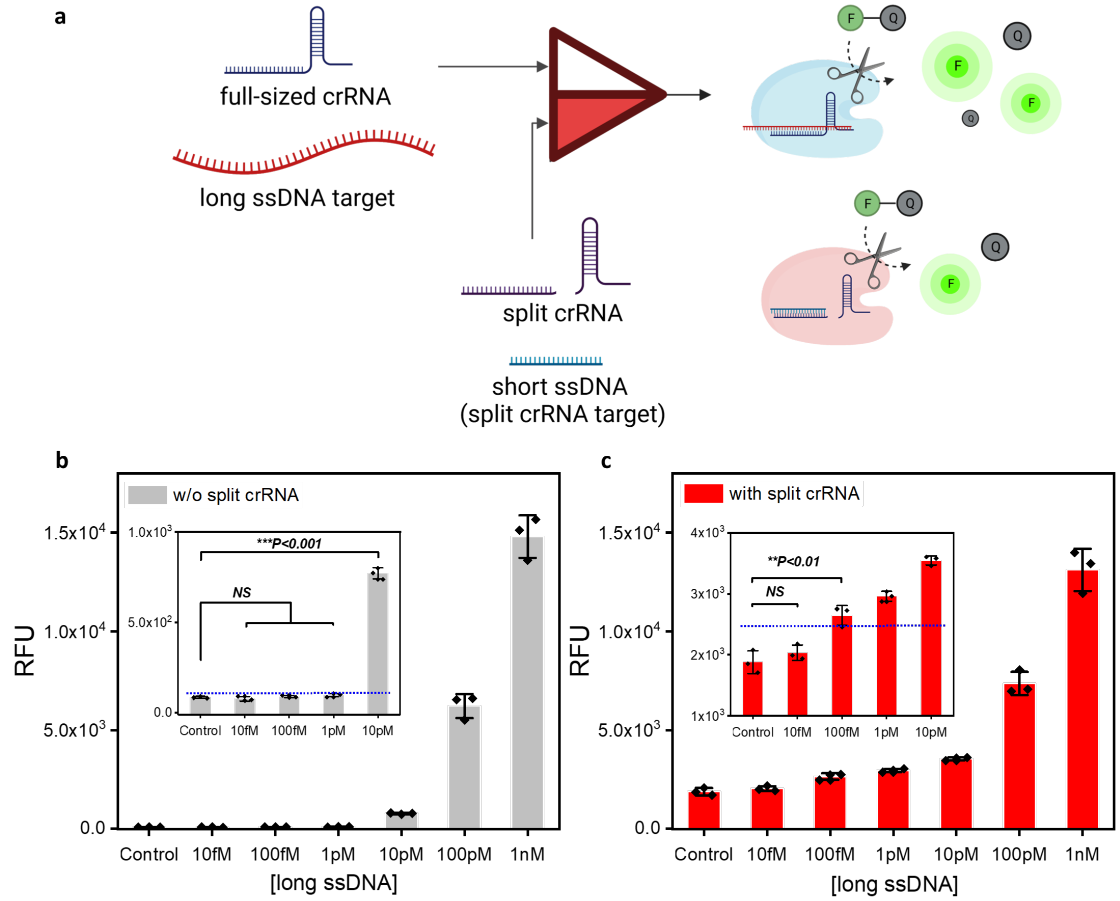

Supplementary Figure 3. (a) Schematic illustration of long ssDNA detection using the asymmetric CRISPR assay. Illustration was created with BioRender.com. **(b, c)** Fluorescence intensity measured after 1 h reaction depending on the concentration of long ssDNA (10 fM ~ 1nM) in the presence and absence of split crRNA. (n = 3, two-tailed Student's *t*-test; NS, not significant (P>0.1); **P<0.01, and ***P<0.001, and data represent mean ± S.D of three technical replicates). The inset blue lines represent the limit of detection (LOD) line. LOD was calculated by the equation, mean blank + 3.3 × S.D of blank. Source data are provided as a Source Data file.

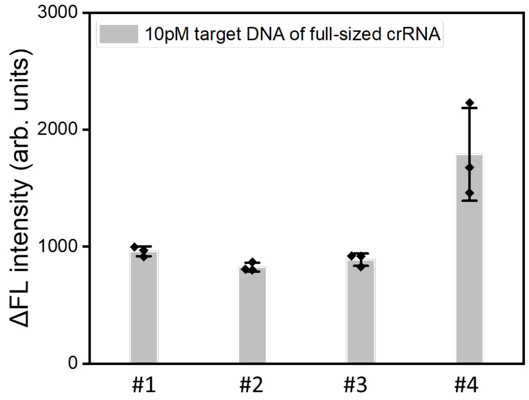

	#1	#2	#3	#4
Cas12a/full-sized crRNA	+	+	+	+
Split crRNA handle	-	+	-	+
Split crRNA spacer	-	-	+	+
Split-T	+	+	+	+

Supplementary Figure 4. **Δ**Fluorescence intensity ($F_{\text{target DNA 10pM}} - F_{\text{control}}$) under different combination of split crRNA handle and spacer. Fluorescence intensity measured after 1 h reaction. (n =3, Data represent means ± S.D of three technical replicates) Source data are provided as a Source Data file.

	RNA target	DNA activator
**a.**	5’ – UGUGCAAAUCUAUGCAAAACUGA	TAGAAGAATGTGTAAGTATAATACGACTCA – 3’
**b.**	5’ – UGUGCAAAUCUAUGCAAAACUGA	TAGAAGAATGTGTAAGTATAATACGACTCA – 3’
**c.**	5’ – UGUGCAAAUCUAUGCAAAACUGA	TAGAAGAATGTGTAAGTATAATACGACTCA – 3’
**d.**	5’ – UGUGCAAAUCUAUGCAAAACUGA	TAGAAGAATGTGTAAGTATAATACGACTCA – 3’
**e.**	5’ – UGUGCAAAUCUAUGCAAAACUGA	TAGAAGAATGTGTAAGTATAATACGACTCA – 3’
**f.**	5’ – UGUGCAAAUCUAUGCAAAACUGA	TAGAAGAATGTGTAAGTATAATACGACTCA – 3’

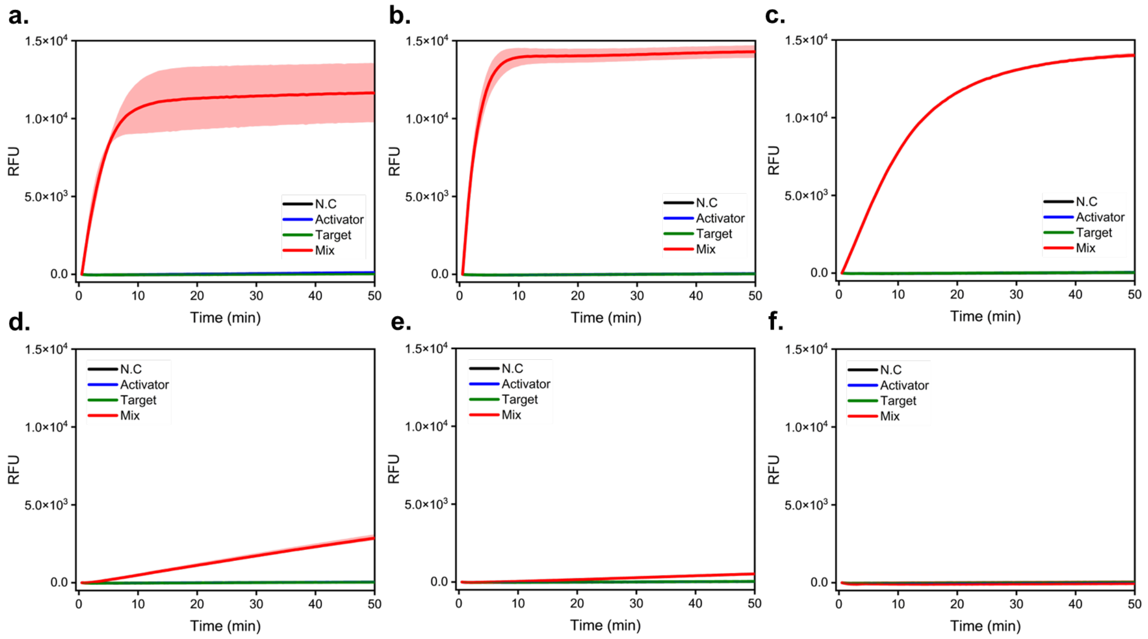

Supplementary Figure 5. Sequences of the ssRNA 5’-targets and ssDNA 3’-activators, locations of the crRNA binding site (upper a-f, red), and corresponding fluorescence signal under various conditions (below a-f, N.C, RNA target, DNA activator, and a mixture of RNA target and DNA activator) [Cas12a] = 100 nM, [RD crRNA] = 10 nM, [DNA activator] = 100 nM, [RNA target] = 10 nM. (n =3, Data represent means ± S.D of three technical replicates) Source data are provided as a Source Data file.

	DNA activator	RNA target
a.	5' – TAGAAGAATGTGTAAGTATAATACGACTCA	UGUGCAAAUCUAUGCAAAACUGA – 3'
b.	5' – TAGAAGAATGTGTAAGTATAATACGACTCA	UGUGCAAAUCUAUGCAAAACUGA – 3'
c.	5' – TAGAAGAATGTGTAAGTATAATACGACTCA	UGUGCAAAUCUAUGCAAAACUGA – 3'
d.	5' – TAGAAGAATGTGTAAGTATAATACGACTCA	UGUGCAAAUCUAUGCAAAACUGA – 3'
e.	5' – TAGAAGAATGTGTAAGTATAATACGACTCA	UGUGCAAAUCUAUGCAAAACUGA – 3'
f.	5' – TAGAAGAATGTGTAAGTATAATACGACTCA	UGUGCAAAUCUAUGCAAAACUGA – 3'

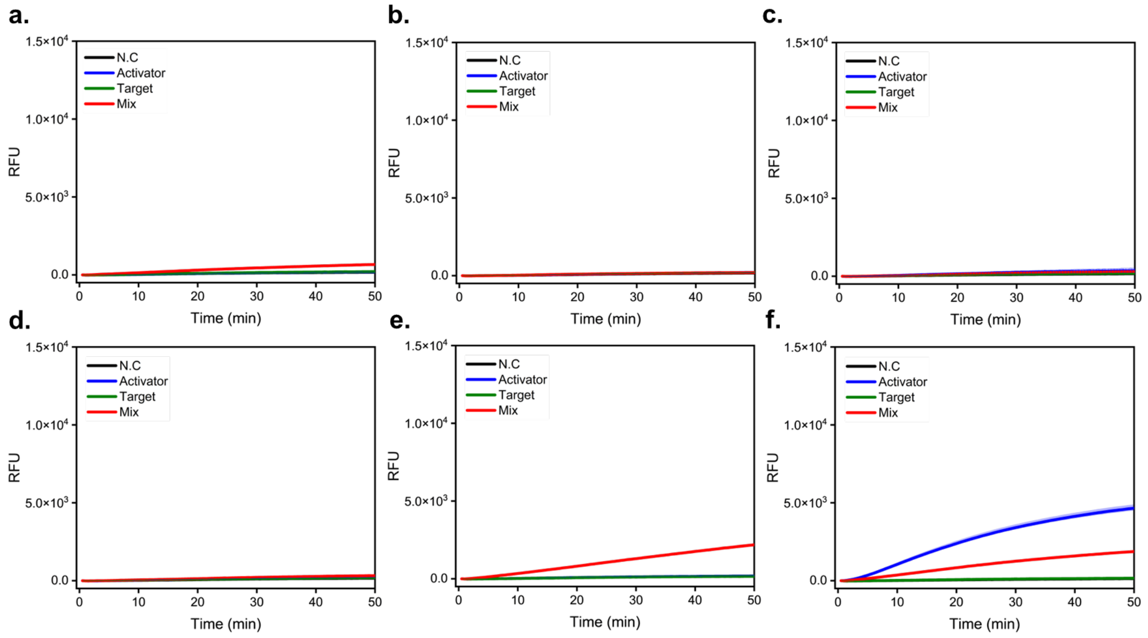

Supplementary Figure 6. Sequences of the ssDNA 5'-activators and ssRNA 3'-targets, locations of the crRNA binding site (upper a-f, red), and corresponding fluorescence signal under various conditions (below a-f, N.C, RNA target, DNA activator, and a mixture of RNA target and DNA activator). [Cas12a] = 100 nM, [DD crRNA] = 10 nM, [DNA activator] = 100 nM, [RNA target] = 100 nM. (n =3, Data represent means ± S.D of three technical replicates) Source data are provided as a Source Data file.

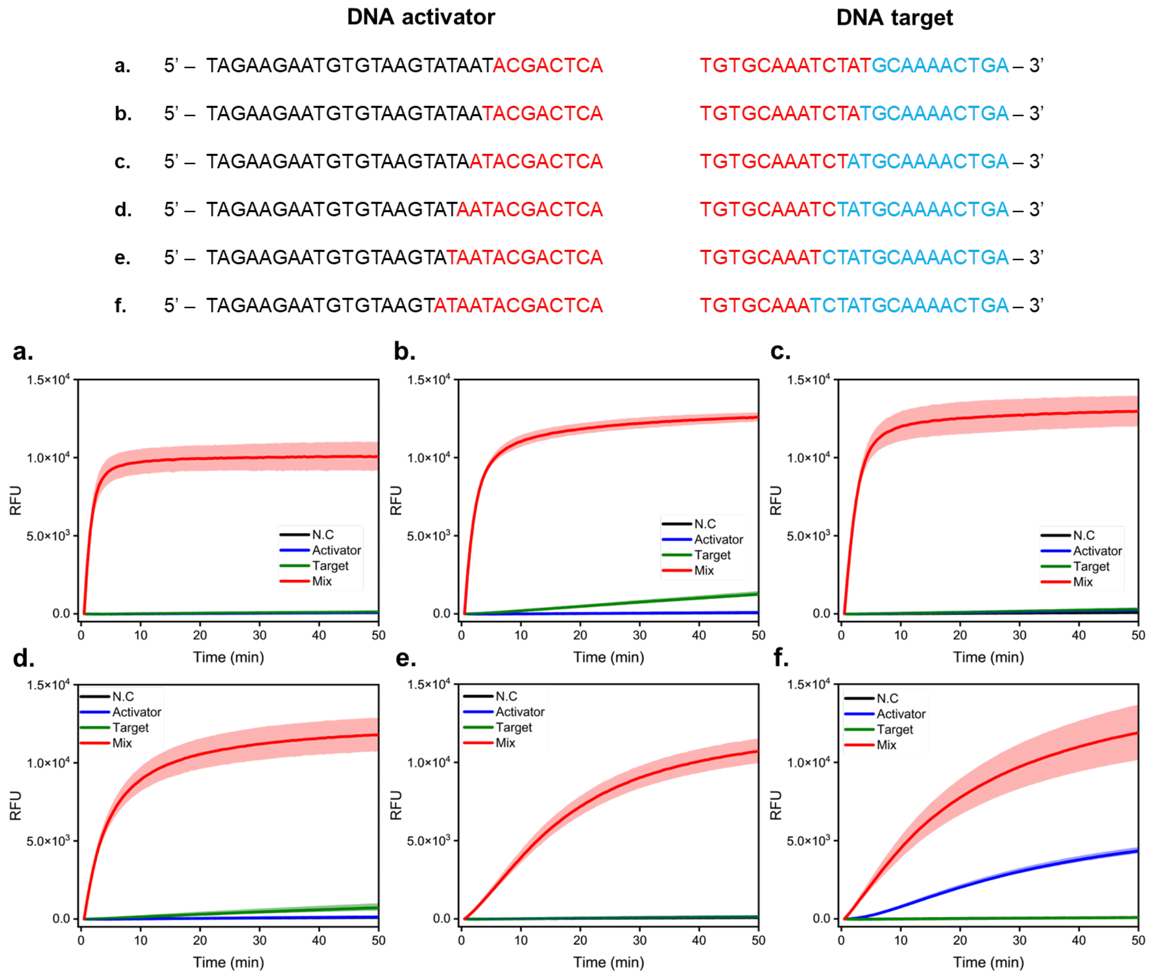

Supplementary Figure 7. Sequences of the ssDNA 5'-activators and ssDNA 3'-targets, locations of the crRNA binding site (upper a-f, red), and corresponding fluorescence signal under various conditions (below a-f, N.C, DNA target, DNA activator, and a mixture of DNA target and DNA activator). [Cas12a] = 100 nM, [DD crRNA] = 10 nM, [DNA activator] = 100 nM, [DNA target] = 100 nM. (n =3, Data represent means ± S.D of three technical replicates) Source data are provided as a Source Data file.

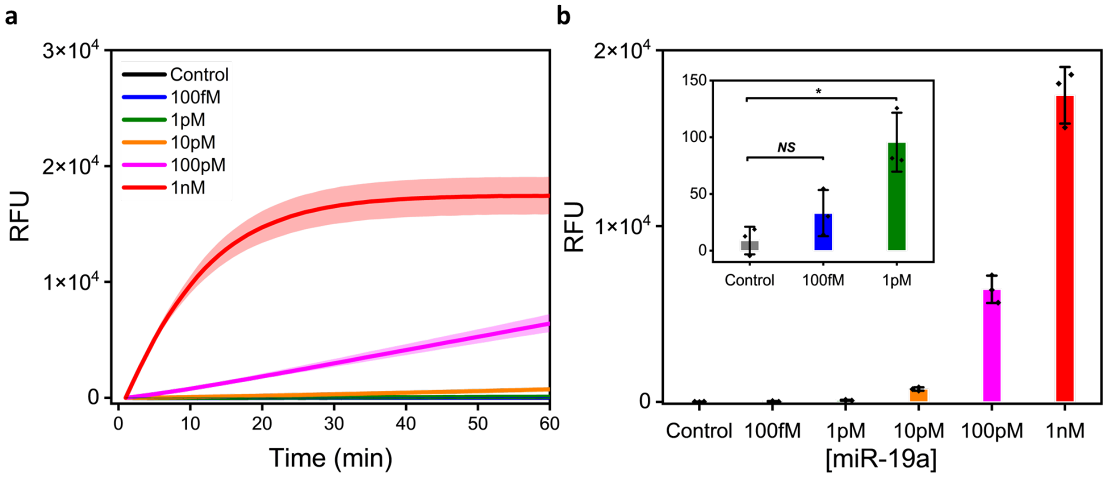

Supplementary Figure 8. **(a)** The real-time fluorescence signal of Cas12a/crRNA reaction using target miR-19a in the concentration range of 100 fM to 1 nM. **(b)** Fluorescence signal measured after 1 h reaction. The limit of detection (LOD) was calculated by the equation, mean blank + 3.3 × S.D of a blank and was estimated to be 1 pM. (n = 3, two-tailed Student's *t*-test; NS, not significant (*P*>0.1); **P*<0.05, data represent mean ± S.D of three technical replicates). Source data are provided as a Source Data file.

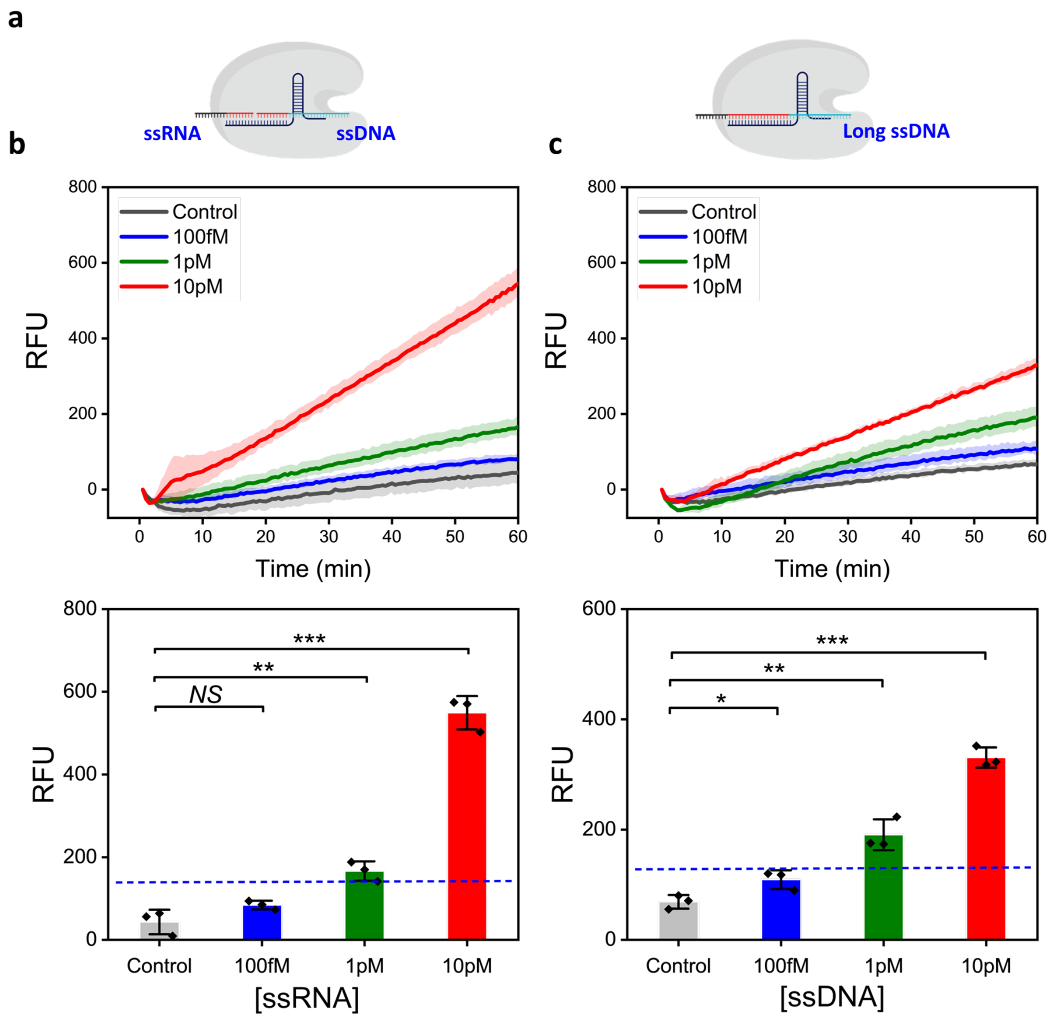

Supplementary Figure 9. Comparison of Cas12a/crRNA *trans*-cleavage activity between a split target (ssRNA and ssDNA) and long ssDNA target containing identical sequences of split ssRNA and ssDNA. **(a)** Schematic illustration of CRISPR detection for split target (ssRNA and ssDNA) and long ssDNA. **(b)** Real-time fluorescence signal and endpoint fluorescence intensity depending on the concentration (100 fM, 1 pM, and 10 pM) of split ssRNA. **(c)** Real-time fluorescence signal and endpoint fluorescence intensity depending on the concentration (100 fM, 1 pM, and 10 pM) of long ssDNA target. Experiments were conducted three times and data represent mean ± S.D of three technical replicates (two-tailed Student's *t*-test; NS, not significant (P>0.1); *P<0.05, **P<0.01, and ***P<0.001). The inset blue lines represent the limit of detection (LOD) line. LOD was calculated by the equation, mean blank + 3.3 × S.D of blank. The LOD of both targets was calculated as 1 pM, respectively. [Cas12a] = 100 nM, [crRNA] = 10nM, and [split ssDNA] = 10 nM. Source data are provided as a Source Data file.

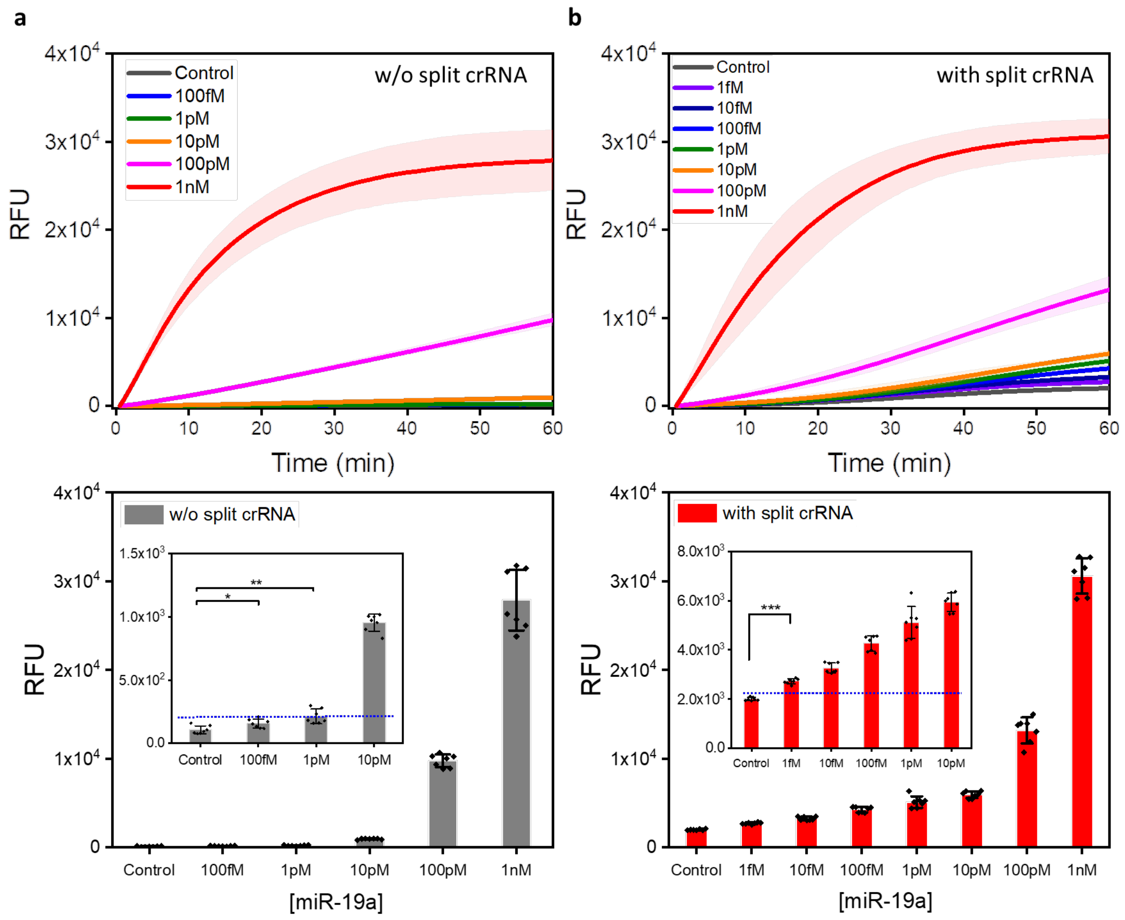

Supplementary Figure 10. Real-time fluorescence signal and the fluorescence intensity measured after 1 h reaction of **(a)** a CRISPR-Cas12a assay without split crRNA and **(b)** the asymmetric CRISPR assay. (n = 7, two-tailed Student's *t*-test; NS, not significant (P>0.1); *P<0.05, **P<0.01, and ***P<0.001; data represent mean ± S.D of seven technical replicates). The inset blue lines represent the limit of detection (LOD) line. LOD was calculated by the equation, mean blank + 3.3 × S.D of blank. Source data are provided as a Source Data file.

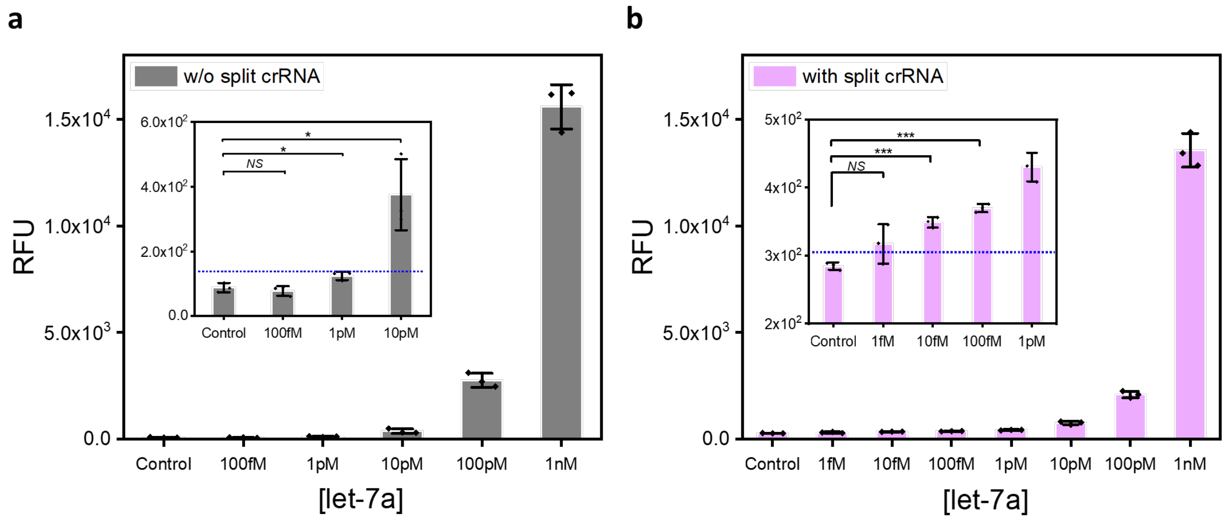

Supplementary Figure 11. The fluorescence signal of let-7a detection using **(a)** a Cas12a/crRNA assay without split crRNA and **(b)** an asymmetric CRISPR assay. The fluorescence signal was measured after 1h incubation. The inset blue lines represent the limit of detection (LOD) line. LOD was calculated by the equation of mean blank + 3.3 × S.D of blank. (n = 3, two-tailed Student's *t*-test; NS, not significant (P>0.1); *P<0.05, **P<0.01, and ***P<0.001; data represent mean ± S.D of three technical replicates) Source data are provided as a Source Data file.

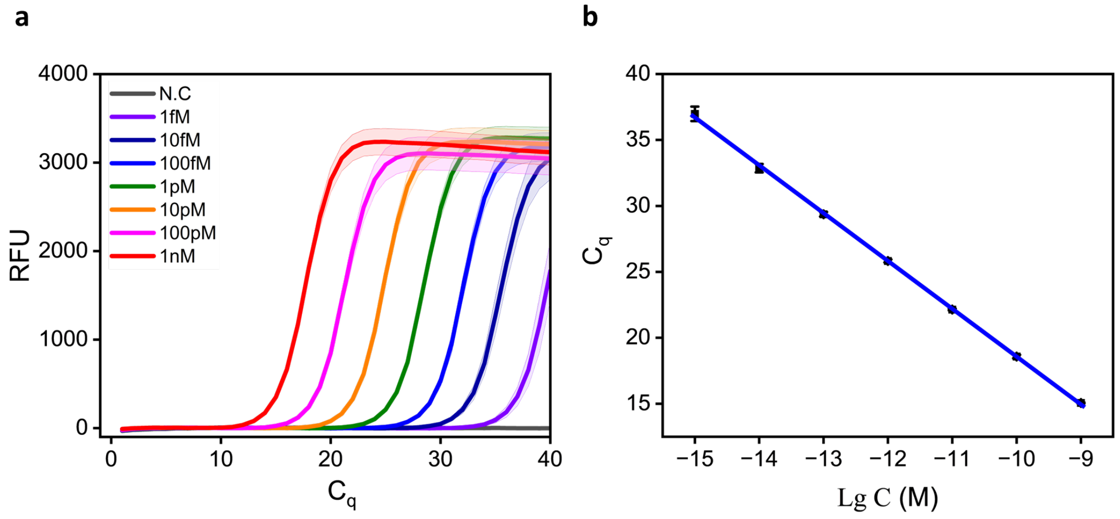

Supplementary Figure 12. (a) Real-time fluorescence responses of standard target miR-19a by RT-qPCR. **(b)** The linear relationship between the Cq and logarithm of miR-19a concentration. The linear regression equation is $y = -3.572x - 17.103$ ($R^2 = 0.999$). n = 3, data represent mean ± S.D of three technical replicates. Source data are provided as a Source Data file.

References

1. Zhong M*, et al.* PCDetection: PolyA-CRISPR/Cas12a-based miRNA detection without PAM restriction. *Biosens Bioelectron* **214**, 114497 (2022).

2. Jia HY, Zhao HL, Wang T, Chen PR, Yin BC, Ye BC. A programmable and sensitive CRISPR/Cas12a-based MicroRNA detection platform combined with hybridization chain reaction. *Biosens Bioelectron* **211**, 114382 (2022).

3. Yan H*, et al.* A one-pot isothermal Cas12-based assay for the sensitive detection of microRNAs. *Nat Biomed Eng*, (2023).

4. Peng S, Tan Z, Chen S, Lei C, Nie Z. Integrating CRISPR-Cas12a with a DNA circuit as a generic sensing platform for amplified detection of microRNA. *Chem Sci* **11**, 7362-7368 (2020).

5. Li X*, et al.* Ultrasensitive detection of microRNAs based on click chemistry-terminal deoxynucleotidyl transferase combined with CRISPR/Cas12a. *Biochimie* **208**, 38-45 (2023).

6. Zhang M, Wang H, Wang H, Wang F, Li Z. CRISPR/Cas12a-Assisted Ligation-Initiated Loop-Mediated Isothermal Amplification (CAL-LAMP) for Highly Specific Detection of microRNAs. *Anal Chem* **93**, 7942-7948 (2021).

7. Zhang G, Zhang L, Tong JT, Zhao XX, Ren JL. CRISPR-Cas12a enhanced rolling circle amplification method for ultrasensitive miRNA detection. *Microchem J* **158**, 105239 (2020).
