# Supplementary material for: Asymmetric CRISPR enabling cascade signal amplification for nucleic acid detection by competitive crRNA
Source: Nat Commun. 2023 Nov 18;14:7504. doi: 10.1038/s41467-023-43389-7 (PMC10657364; doi:10.1038/s41467-023-43389-7)
Supplement: Supplementary file 2 — Reporting Summary [file 41467_2023_43389_MOESM2_ESM.pdf]

nature portfolio

Corresponding author(s): Changchun Liu
Last updated by author(s): Nov 1, 2023

Reporting Summary

Nature Portfolio wishes to improve the reproducibility of the work that we publish. This form provides structure for consistency and transparency in reporting. For further information on Nature Portfolio policies, see our Editorial Policies and the Editorial Policy Checklist.

Statistics

For all statistical analyses, confirm that the following items are present in the figure legend, table legend, main text, or Methods section.

n/a	Confirmed	
☐	☒	The exact sample size (*n*) for each experimental group/condition, given as a discrete number and unit of measurement
☐	☒	A statement on whether measurements were taken from distinct samples or whether the same sample was measured repeatedly
☐	☒	The statistical test(s) used AND whether they are one- or two-sided<br>*Only common tests should be described solely by name; describe more complex techniques in the Methods section.*
☐	☒	A description of all covariates tested
☐	☒	A description of any assumptions or corrections, such as tests of normality and adjustment for multiple comparisons
☐	☒	A full description of the statistical parameters including central tendency (e.g. means) or other basic estimates (e.g. regression coefficient) AND variation (e.g. standard deviation) or associated estimates of uncertainty (e.g. confidence intervals)
☐	☒	For null hypothesis testing, the test statistic (e.g. *F*, *t*, *r*) with confidence intervals, effect sizes, degrees of freedom and *P* value noted<br>*Give P values as exact values whenever suitable.*
☒	☐	For Bayesian analysis, information on the choice of priors and Markov chain Monte Carlo settings
☒	☐	For hierarchical and complex designs, identification of the appropriate level for tests and full reporting of outcomes
☒	☐	Estimates of effect sizes (e.g. Cohen's *d*, Pearson's *r*), indicating how they were calculated

Our web collection on statistics for biologists contains articles on many of the points above.

Software and code

Policy information about availability of computer code

Data collection	Data collection is described in the Methods section. Real-time fluorescence was determined using Bio-Rad CFX96 Touch Real-time PCR Detection System. The endpoint fluorescence was the raw fluorescence determined by the Bio-Rad CFX96 Touch Real-time PCR Detection system. The gel imaging was conducted using Bio-Rad ChemiDoc Imaging System with its built-in UV and fluorescence channel.
Data analysis	Data analysis is described in the Methods section. Fluorescence data was analyzed using Microsoft Excel (version 2302) and graphs were plotted using OriginPro version 9.6. Statistical analysis was done in Microsoft Excel version 2302. Gel images were analyzed using Image J. Probit analysis was done in MedCalc statistical software.

For manuscripts utilizing custom algorithms or software that are central to the research but not yet described in published literature, software must be made available to editors and reviewers. We strongly encourage code deposition in a community repository (e.g. GitHub). See the Nature Portfolio guidelines for submitting code & software for further information.

Data

Policy information about availability of data

All manuscripts must include a data availability statement. This statement should provide the following information, where applicable:

- Accession codes, unique identifiers, or web links for publicly available datasets
- A description of any restrictions on data availability
- For clinical datasets or third party data, please ensure that the statement adheres to our policy

Data supporting the findings of this study are available within the article and its Supplementary Figures. Source data are available in the Source Data file. Additional

details on datasets and protocols that support the findings of this study will be made available by the corresponding author upon reasonable request.

Research involving human participants, their data, or biological material

Policy information about studies with human participants or human data. See also policy information about sex, gender (identity/presentation), and sexual orientation and race, ethnicity and racism.

Reporting on sex and gender	The information was provided in supplementary Table 5.
Reporting on race, ethnicity, or other socially relevant groupings	N/A
Population characteristics	N/A
Recruitment	N/A
Ethics oversight	The study was approved by ethics committee at the University of Connecticut Health Center.

Note that full information on the approval of the study protocol must also be provided in the manuscript.

Field-specific reporting

Please select the one below that is the best fit for your research. If you are not sure, read the appropriate sections before making your selection.

- [x] Life sciences
- [ ] Behavioural & social sciences
- [ ] Ecological, evolutionary & environmental sciences

For a reference copy of the document with all sections, see nature.com/documents/nr-reporting-summary-flat.pdf

Life sciences study design

All studies must disclose on these points even when the disclosure is negative.

Sample size	Sample sizes were determined based on previous publications related to the study, attempting to have a minimum of n = 3 technical replicates with sufficient reproducibility.
Data exclusions	No data exclusions
Replication	All experiments were carried out with at least three replicates. Attempts of data replication were successful.
Randomization	All the human plasma samples were randomly chosen. Because in this study, we only describe proof-of-concept testing of miRNA analysis in plasma levels.
Blinding	Data collection and analysis were not blinded. Determination of concentrations is considered an objective measure, not subject to bias and therefore the integrity of the results are not impacted when running the study and analysis unblinded.

Reporting for specific materials, systems and methods

We require information from authors about some types of materials, experimental systems and methods used in many studies. Here, indicate whether each material, system or method listed is relevant to your study. If you are not sure if a list item applies to your research, read the appropriate section before selecting a response.

Materials & experimental systems

n/a	Involved in the study	
x		Antibodies
x		Eukaryotic cell lines
x		Palaeontology and archaeology
x		Animals and other organisms
x		Clinical data
x		Dual use research of concern
x		Plants

Methods

n/a	Involved in the study	
x		ChIP-seq
x		Flow cytometry
x		MRI-based neuroimaging
